# Dominant influenza A(H3N2) and B/Yamagata virus circulation in EU/EEA, 2016/17 and 2017/18 seasons, respectively

**DOI:** 10.2807/1560-7917.ES.2018.23.13.18-00146

**Published:** 2018-03-29

**Authors:** Cornelia Adlhoch, René Snacken, Angeliki Melidou, Silviu Ionescu, Pasi Penttinen, the European Influenza Surveillance Network

**Affiliations:** 1 European Centre for Disease Prevention and Control (ECDC), Stockholm, Sweden; 2 Microbiology Department, School of Medicine, Aristotle University of Thessaloniki, Thessaloniki, Greece; 3 The members of the European Influenza Surveillance Network are listed at the end of article

**Keywords:** Europe, influenza, surveillance, epidemiology, severity, mortality, intensive care

## Abstract

We use surveillance data to describe influenza A and B virus circulation over two consecutive seasons with excess all-cause mortality in Europe, especially in people aged 60 years and older. Influenza A(H3N2) virus dominated in 2016/17 and B/Yamagata in 2017/18. The latter season was prolonged with positivity rates above 50% among sentinel detections for at least 12 weeks. With a current west–east geographical spread, high influenza activity might still be expected in eastern Europe.

The yearly influenza epidemics during each winter season vary in burden and severity. During the 2016/17 and 2017/18 seasons, all-cause excess mortality was observed during periods of high influenza virus circulation [1,2]. Our aim is to describe and compare the pattern of influenza virus circulation and related disease severity by number of patients and fatal cases in intensive care units (ICUs) across European Union/European Economic Area (EU/EEA) countries for the seasons 2016/17 and 2017/18. As influenza circulation progressed from a west to east direction across Europe in 2017/18, a better understanding of the current epidemiological situation might help to prepare countries in the eastern part of the World Health Organization (WHO) European Region for high influenza activity and severity [3].

## Influenza surveillance in Europe

The European Influenza Surveillance Network (EISN) performs influenza surveillance from week 40 to week 20 of the following year [4,5]. Weekly epidemiological and virological influenza data are collected from 30 EU/EAA countries and 11 countries report data on severe and fatal cases with laboratory-confirmed influenza in ICUs. Collected data in selected primary care settings include the percentage of these sentinel specimens testing positive for influenza [6]. 

## Influenza virus circulation in the 2016/17 and 2017/18 seasons

**Figure 1:**
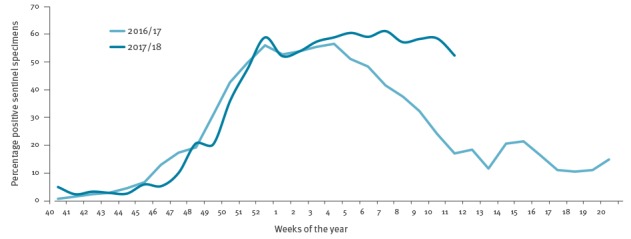
Percentage positivity for influenza viruses among sentinel specimens, by week and season, European Union/European Economic Area, 2016/17 and 2017/18 (up to week 11)

The influenza virus positivity rates among sentinel specimens in EU/EEA countries over the 2016/17 and 2017/18 seasons are shown in Figure 1.

### 2016/17 seasonal influenza epidemic

#### Sentinel surveillance outcome

**Figure 2:**
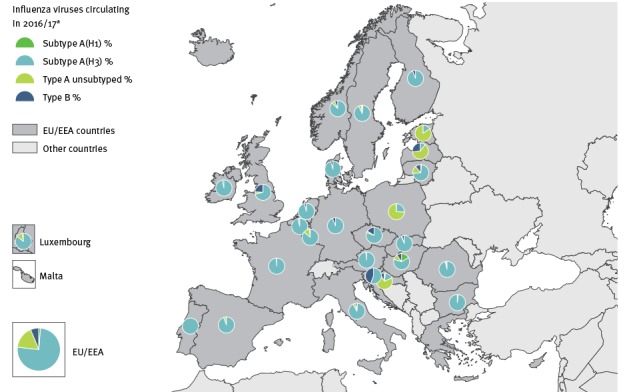
Distribution of viral types/subtypes in sentinel specimens by country, European Union/European Economic Area, influenza season 2016/17

The 2016/17 epidemic was characterised by an early start, with overall sentinel detections crossing the 10% positivity threshold in week 46/2016 (Figure 1) and with a dominance (75%; 10,242/13,584) of influenza A(H3N2) viruses among all positive sentinel specimens, 17% (n = 2,318) influenza A unsubtyped, 1% (n = 172) A(H1N1)pdm09, 2% (n = 281) B no lineage, 1% (n = 117) B/Victoria and 3% (n = 454) B/Yamagata lineage) [7]. Only Slovenia reported a substantial proportion (44%; 113/259) of B/Yamagata viruses in sentinel specimens (Figure 2). The positivity rate in the EU/EEA remained above 10% for 27 consecutive weeks with a dominance of B/Yamagata viruses in the final weeks of the season. The peak phase with positivity levels above 50% (maximum positivity: 57%) lasted for 6 consecutive weeks.

EU/EEA: European Union/European Economic Area.

^a^ Only sentinel specimens are included.

#### Results from surveillance in intensive care units

**Figure 3:**
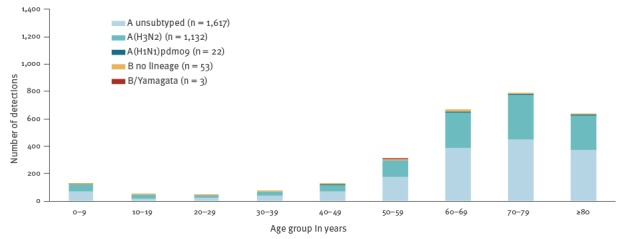
Distribution of laboratory-confirmed influenza cases admitted to ICU, by age group and type/subtype, in the reporting European Union/European Economic Area countries, 2016/17 season (n = 2,827 patients)^a^

During 2016/17, 10 countries (Czech Republic, Denmark, Finland, France, Ireland, Romania, Slovakia, Spain, Sweden, and the United Kingdom (UK)) reported a total of 3,959 patients admitted to ICUs with laboratory-confirmed influenza (Figure 3). Of these patients, 96% (n = 3,813) were infected with influenza A virus, with 92% (1,465/1,592) of the subtyped A viruses being A(H3N2). The majority of cases (81%; 3,194/3,959) were reported from three countries, namely France (n = 1,469 cases), the UK (n = 1,109) and Spain (n = 616). Most cases in the 2016/17 season occurred up to week 11, by which 97% (3,837/3,959) of all the season’s cases in ICU and 99% (593/599) of all fatal cases had been reported.

ICU: intensive care unit.

^a^ Of the total reported 3,959 patients, 2,827 where age information was available are included in the figure.

### 2017/18 seasonal influenza epidemic

#### Sentinel surveillance outcome

During the 2017/18 season, the influenza positivity of tested sentinel specimens passed the 10% threshold in week 47 2017, and the 50% threshold in week 52 2017. Positivity rates exceeded 52% for over 12 weeks indicating an unusually protracted peak period of influenza activity across the countries (maximum positivity: 61%; Figure 1). In the previous seven seasons, the number of weeks with a positivity of 50% and higher ranged between 0 (2009/10; 2013/14) and 8 (2012/13; 2015/16) weeks.

**Figure 4:**
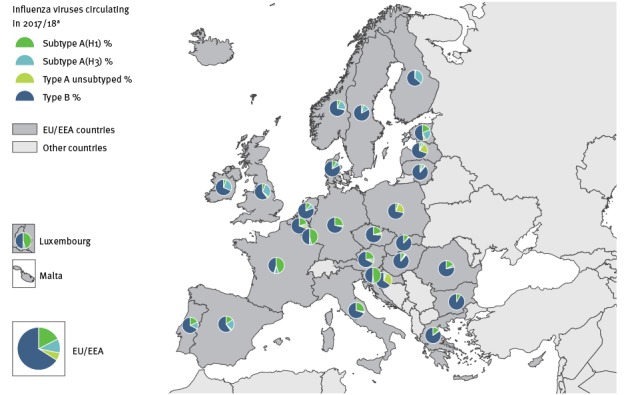
Distribution of viral types/subtypes in sentinel specimens by country, European Union/European Economic Area, influenza season 2017/18 (up to week 11)

During the initial weeks of the 2017/18 season, similar numbers of influenza A and B viruses were detected in sentinel specimens, but between weeks 48 2017 and 11 2018, influenza B viruses were dominant (66%; 12,455/18,888). Among B viruses with lineage determination, 97% (5,687/5,860) were of the Yamagata lineage. Overall during this period, 6% (n = 1,102) unsubtyped influenza A, 17% (n = 3,283) A(H1N1)pdm09, 11% (n = 2,048) A(H3N2), 35% (n = 6,595) B without lineage determination, 1% (n = 173) B/Victoria and 30% (n = 5,687) B/Yamagata lineage were detected. All countries had a proportion of sentinel B viruses of 46% or higher. Both influenza A(H1N1)pdm09 and A(H3N2) viruses co-circulated with B viruses, but the pattern and magnitude varied across countries. These different patterns are more visible at country-level (Figure 4). 

EU/EEA: European Union/European Economic Area.

^a^ Only sentinel specimens are included.

#### Results from surveillance in intensive care units 

**Figure 5:**
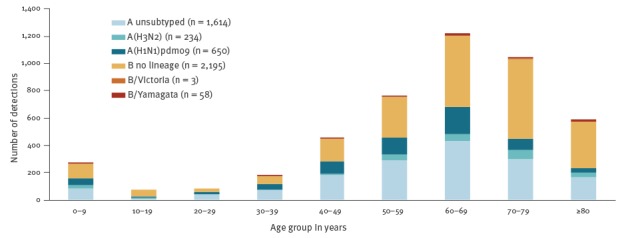
Distribution of laboratory-confirmed influenza cases admitted to ICU, by age group and type/subtype, in the reporting European Union/European Economic Area countries, 2017/18 season (up to week 11) (n = 4,754)^a^

^a^ Of the total reported 7,789 patients, 4,754 where age information was available are included in the figure.

Up to week 11 2018, 7,789 laboratory-confirmed influenza cases in ICUs were reported from 10 countries (Czech Republic, Denmark, Finland, France, Ireland, the Netherlands, Romania, Spain, Sweden, and the UK). Altogether, the UK (n = 2,983), France (n = 2,614) and Spain (n = 1,119) reported the vast majority of cases (86%; 6,716/7,789). Comparing data up to week 11 in 2017/18 to the complete 2016/17 season, this is an increase of ICU cases by 169%, 78% and 82% in these countries, respectively (Figure 3, Figure 5).

Influenza A was detected in 52% (n = 4,036) of the 7,789 patients, and 59% (n = 872) of the 1,471 subtyped influenza A viruses were reported as A(H1N1)pdm09 with 51% (n = 449) of them reported by France.

The vast majority (81%; 190/234) of all B viruses as well as A(H3N2) viruses were detected in ICU patients aged 50 years and older. The majority (64%; 419/650) of patients infected with A(H1N1)pdm09 were 40 to 69 years-old.

## Disease severity in 2016/17 and 2017/18

The severity of disease caused by B viruses during the 2017/18 season is also reflected in the high proportion of B infections among fatal outcomes reported from ICUs (49%; 420/851). This is in contrast with the previous season in which 1% (5/599) of fatal cases in ICUs died of influenza B infection. In 2016/17, 0.5% (3/599) of the fatal cases were infected with A(H1N1)pdm09, 44% (266/599) with A(H3N2) and 54% (325/599) with an unsubtyped influenza A virus. In 2017/18, 15% (124/851) of the fatal cases were infected with A(H1N1)pdm09, 5% (42/851) with A(H3N2) and 31% (265/851) with an unsubtyped influenza A virus. 

Among all cases reported with influenza B infection in ICUs and known outcome, 5/52 (10%) died in 2016/17, while 420/1,983 (21%) died in 2017/18. More than 50% (333/636) of all fatal cases over 60 years of age had influenza B and among fatal cases infected with influenza B virus, 79% (333/420) were 60 years of age and older. A similar distribution between virus type and age has been also observed in the B/Yamagata virus dominated season 2012/13.

## Discussion

The ongoing 2017/18 influenza season with dominant circulation of influenza B and a co-circulation of A(H1N1)pdm09 and/or A(H3N2) viruses is proving to have a severe impact, as did the 2016/17 season with its dominance of influenza A(H3N2). The dominance of A(H3N2) viruses associated with a high severity, prolonged season and all-cause excess mortality in the EU/EEA in 2016/17 [2] is comparable to the situation in the United States during the 2017/18 season [8]. The dominant B/Yamagata virus circulation with mixed A virus pattern in 2017/18 causing high severity, long duration of peak influenza activity and all-cause excess mortality in the EU/EEA is comparable to 2012/13, an influenza B/Yamagata virus dominated season with A(H1N1)pdm09 and A(H3N2) co-circulation [1,2,9,10]. Influenza A(H3N2) viruses are known to affect disproportionately those aged above 65 years, leading to outbreaks in long-term care homes, increases in hospitalisation and mortality in this age group [11,12]. B viruses are described as causing milder disease and affecting more the younger age groups [13]. In both seasons described here, however, we observed the highest number of severe cases in patients admitted to ICUs aged 60 years and older. As seen previously, patients infected with A(H1N1)pdm09 in ICU were slightly younger than patients infected with influenza A(H3N2) or B [14,15]. 

The reason for the prolonged and increased activity as well as the severe clinical impact of influenza B viruses during the 2017/18 season is not fully understood. Countries perform either sentinel or universal hospital surveillance and no major change in reporting has occurred which would explain the increase of cases in ICU [16].

Although the most commonly used trivalent vaccine contained the B/Victoria lineage in both 2016/17 and 2017/18 seasons, a moderate vaccine effectiveness for B/Yamagata and A(H1N1)pdm09 in 2017/18 was observed; the effectiveness for A(H3N2) viruses in both seasons, however, was low [12,17]. The moderate and low vaccine effectiveness as well as low vaccine coverage might have contributed to the lower protection in the population together with an accumulation of susceptible people since the last dominant B/Yamagata circulation five seasons ago [18]. Further in-depth analyses are needed to describe the mostly affected population and identify relevant underlying co-morbidities and other factors, e.g. cold weather, that might have contributed to the prolonged virus activity and severity. Neuraminidase inhibitors remain an option for the prophylaxis and treatment of the currently circulating influenza viruses, and their use should be considered along the national and international guidance and recommendations, especially for cases with severe and rapidly progressing disease [19]. The season in Europe has progressed in a marked west–east direction and countries in the eastern part of the WHO European Region should be prepared for possible cases of severe disease and impact on healthcare services. However, influenza activity remains high also in central and western parts of Europe with continuously observed all-cause mortality.

## Notes

**Members of the European Influenza Surveillance Network:** WHO: Piers Mook, Tamara Meerhoff; Austria: Theresia Popow-Kraupp, Daniela Schmid; Belgium: Barbezange Cyril, Bossuyt Nathalie, Moreels Sarah, Thomas Isabelle, Van Casteren Viviane; Bulgaria: Korsun Neli; Croatia: Draženović Vladimir; Cyprus: Koliou Maria, Pieridou Despo; Czech Republic: Havlíčková Martina, Jiřincová Helena, Kynčl Jan, Denmark: Fischer Thea Kølsen, Krause Knudsen Lisbet, Trebbien Ramona; Estonia: Päll Kaie, Sadikova Olga; Finland: Haveri Anu, Ikonen Niina, Lyytikäinen Outi, Murtopuro Satu; France: Behillil Sylvie, Bernard-Stoecklin Sibylle, Blanchon Thierry, Campese Christine, Enouf Vincent, Lina Bruno, Turbelin Clement, Valette Martine, van der Werf Sylvie; Germany: Buda Silke, Dürrwald Ralf; Greece: Kalkouni Ourania, , Mentis Andreas, Papa Anna, Pogka Vasiliki; Hungary: Jankovics István, Molnár Zsuzsanna, Rózsa Mónika; Iceland: Löve Arthur, Sigmundsdóttir Guðrún; Ireland: Connell Jeff, Domegan Lisa, Duffy Margaret, Dunford Linda, O’Donnell Joan; Italy: Bella Antonino, Rizzo Caterina, Simona Puzelli, Maria Rita Castrucci; Latvia: Nikiforova Raina, Pakarna Gatis, Zamjatina Natalija; Liechtenstein: Jamnicki Abegg Marina; Lithuania: Griškevičius Algirdas, Lipnickienė Vilnelė, Muralytė Svajūnė; Luxembourg: Fournier Guillaume, Nguyen Trung; Malta: Barbara Christopher, Maistre Melillo Jackie, Melillo Tanya, Zahra Graziella; The Netherlands: de Lange Marit, Dijkstra Frederika, Donker Ge, Fouchier Ron, Hooiveld Mariette, Marbus Sierk, Meijer Adam, Teirlinck Anne; Norway: Bragstad Karoline, Dudman Susanne Gjeruldsen, Hauge Siri Helene, Hungnes Olav, Tønnessen Ragnhild; Poland: Brydak Lidia, Cieślak Katarzyna, Zielinski Andrzej; Portugal: Figueiredo Augusto Gonçalo, Machado Jorge, Moreira Guiomar Raquel, Nogueira Paulo, Rebelo De Andrade Helena, Rodrigues Ana Paula; Romania: Ivanciuc Alina, Odette Popovici, Popescu Rodica; Slovakia: Staronová Edita, Mikas Ján; Slovenia: Berginc Nataša, Prosenc Katarina, Sočan Maja, Grilc Eva; Spain: Casas Inmaculada, Delgado Sanz Concha, Larrauri Amparo, Marcos María Angeles, Oliva Jesus, Ortiz De Lejarazu Leonardo Raul, Pozo Francisco, Vega Tomas; Sweden: Mia Brytting, AnnaSara Carnahan; United Kingdom: Coyle Peter, Daniels Rodney Stuart, Gunson Rory, Kearns Cathriona, MacLean Alasdair, McCaughey Conall, McMenamin Jim, Moore Catherine, Nugent Christopher, Pebody Richard, Phin Nick, Potts Alison, Reynolds Arlene, Shaw Primrose Louise, Zambon Maria.
